# Supplementary material for: Content-rich biological network constructed by mining PubMed abstracts
Source: BMC Bioinformatics. 2004 Oct 8;5:147. doi: 10.1186/1471-2105-5-147 (PMC528731; doi:10.1186/1471-2105-5-147)
Supplement: Additional File 2 — The original results of the above study (non-essential files are deleted to keep the file size under the limit set by BMC bioinformatics). [file 1471-2105-5-147-S2.bz2 › chilibotAdditionalFile2/dip05/77ID9144171E288/html/ABL2_RIN1.html]

 


 **ABL2** and **RIN1** 
  
Found 9 abstracts in PubMed, retrieved 05.  
 

 What does Google say? 
 PDF only 
| .edu only 

---

**Interactive relationship** (e.g. stimulation, inhibition, etc)

**Inhibitory relationship**- However, RIN  [ **RIN1** ]  Arg  [ **ABL2** ]  972 showed a significant decrease in binding of the p85 subunit of phosphatidylinositol 3 kinase PI 3 kinase with IRS 1, compared with RIN  [ **RIN1** ]  WT.  Ref: 10430617 J Clin Invest, 1999
**Neutral relationship**- Calcium dependent benzyloxycarbonyl Arg  [ **ABL2** ]  Ser Lys Arg  [ **ABL2** ]  aminomethylcoumarin cleaving activity was found in spent Rin  [ **RIN1** ]  cell medium.  Ref: 8227053 J Biol Chem, 1993

**Non-interactive relationship** (e.g. studied together, co-existance, homology, etc.)

- In this study, we stably overexpressed both wild type IRS 1 RIN  [ **RIN1** ]  WT and Arg  [ **ABL2** ]  972 IRS 1 variant RIN  [ **RIN1** ]  Arg  [ **ABL2** ]  972 in RIN  [ **RIN1** ]  beta cells to investigate directly whether the polymorphism in codon 972 of IRS 1 impairs insulin secretion.  Ref: 10430617 J Clin Invest, 1999
- By contrast, RIN  [ **RIN1** ]  cells expressing Arg  [ **ABL2** ]  972 IRS 1 exhibited a marked decrease in both glucose and sulfonylurea stimulated insulin secretion compared with RIN  [ **RIN1** ]  WT.  Ref: 10430617 J Clin Invest, 1999
- Compared with control RIN  [ **RIN1** ]  cells, insulin content was reduced to the same extent in RIN  [ **RIN1** ]  WT or RIN  [ **RIN1** ]  Arg  [ **ABL2** ]  972 at both the protein and mRNA levels.  Ref: 10430617 J Clin Invest, 1999
